# The Influence of Pubertal Development on Adolescent Depression: The Mediating Effects of Negative Physical Self and Interpersonal Stress

**DOI:** 10.3389/fpsyt.2021.786386

**Published:** 2021-11-18

**Authors:** Lijiao Jiang, Dandan Yang, Yitong Li, Jiajin Yuan

**Affiliations:** ^1^The Affective Cognition and Regulation Laboratory, Institute of Brain and Psychological Sciences, Sichuan Normal University, Chengdu, China; ^2^Department of Psychology, Wuhan University, Wuhan, China; ^3^Faculty of Psychology, Southwest University, Chongqing, China; ^4^School of Psychology, Northwest Normal University, Lanzhou, China

**Keywords:** adolescent development, depression, negative physical self, interpersonal stress, academic self

## Abstract

The current study examined the influence of pubertal development stage on depression and its psychosocial mechanisms in a non-clinical population of 502 adolescents (244 boys and 258 girls) in China, graded 5 to 8. Results indicated that (1) pubertal development was positively correlated with depression, negative physical self and interpersonal stress. (2) There is a significant gender by pubertal development interaction on the measure of academic self-concept, which is accounted for by decreased academic self in boys but not in girls as a function of pubertal development. (3) Mediation analyses show that increased depression in late compared to pre- puberty is partly mediated by the enhancement of negative physical self and interpersonal stress. These findings suggest that the late stage of puberty is coupled by a higher risk of depression in adolescents partly through increased negative physical self and interpersonal stress.

## Introduction

Adolescent depression is a common neuropsychiatric disorder. Epidemiological studies have shown that the onset of depression peaks in adolescence (1–3), and lifelong depression usually begins at puberty (4). Early studies of adolescent health showed that the incidence of depressive symptoms increases significantly after the age of 12 (5), and levels of depressive disorders were significantly higher in mid adolescence than in childhood (6, 7). Adolescent depression can affect normal growth, cause serious educational barriers, and impair social relationships with peers and families (8–12). In addition, adolescent depression is closely associated with adult depression, suicide ideation and behavior (12, 13). There are also gender differences in adolescent depression. Studies have shown that pre-pubertal boys have a higher proportion of depressive symptoms than counterpart girls (14), however, with the development of puberty, the depression level of girls gradually increases with their developmental stage, especially by ages 12 to 13 or in the middle of puberty (15). In brief, previous researches have suggested that compared with childhood, the risk of adolescent depression increases, with greater prevalence in girls than in boys.

Prior studies have proposed several explanations for this phenomenon (16–18), First, physiological changes associated with adolescent development may have an impact on adolescent depression. Adolescents need to face sudden physical changes in the onset of puberty. Rapid changes in hormone levels and other physiological characteristics bring emotional stress to adolescents and even result in adaptive disorders (e.g., depression, binge drinking) (2, 18, 19). This adaptive stress may vary at different pubertal stages. For example, depression in pre-pubertal children is less common than that in adolescents (6, 7, 20), while the rate of depression increased significantly in the mid-puberty (16). Girls have a higher rate of depression after Tanner III and boys have a higher rate of depression before that (14).

Secondly, adolescent development is also linked with multiple psychosocial stressors, such as physical self-dissatisfaction, interpersonal stress and academic stress (17, 21–23). Several studies have shown that negative physical self (17, 24–26), interpersonal stress (27, 28), and academic self-stress (29) all contribute to the increase in adolescent depression.

In more detail, studies have indicated that body image dissatisfaction can be especially harmful to adolescents who undergo dramatic physical changes (24). In addition, the quality of parent-child relationship declines temporarily and the conflict increases with adolescent development (30). Indirect peer stress (e.g., the emergence of school and class norms) increases from early to middle adolescence (27). Academic stress also increases with adolescent ages, as academic pressure is indicated to be one of the most common sources of stress for teenagers (29, 31). In summary, adolescence development is associated with adaptive stresses that are considered to contribute to depressive disorders.

However, pubertal development is the key event during adolescence (32). Epidemiological evidence has shown that the risk of psychosocial disorders starts to rise with pubertal transition, including depression and behavioral disorders like substance misuse (3). Nonetheless, most studies of adolescent development used age or grade as the criteria to define the timing of adolescence (33–35), with the impacts of puberty on depression and psychosocial stresses less studied. As we know, age and grade are not necessarily indicative of levels of physical maturation, which, by contrast, is embodied by pubertal developmental stage.

In this regard, despite current knowledge of the relation between adolescent development and adaptive stress or depression, it is necessary to determine how puberty, the land marking event of adolescence, influences depression in teenagers. As analyzed above, it is possible that psychosocial stressors, from body image concern, interpersonal stress to academic stress, may mediate the association between puberty and depression. However, the roles of these psychosocial variables in the association between pubertal development and depression need to directly illustrated.

In addition, prior studies have indicated sex difference in the prevalence of depression and certain psychosocial stress (e.g., interpersonal stress) during adolescence (16, 36, 37). Also, there is evidence that the pattern of sex difference in emotion processing varies as a function of pubertal stage (38). Therefore, this study first examined sex differences in the impact of pubertal development on depression and perceived psychosocial stress. Then, according to the result, we further explored psychosocial factors that may mediate the relationship between pubertal development and adolescent depression, taking sex as a moderator. The mediation effect of each single stressor as well as the comprehensive effects of multiple stressors were examined, respectively.

## Methods

### Participants

Five hundred and two (*N* = 502) teenagers from grade 5 to grade 8 in Chengjiang Primary School and Chongqing 23 th high school in China were recruited by random sampling. Among them (244 boys and 258 girls), 60 (M_age_ = 11.23, SD = 0.93) were classified as in the pre-pubertal, 145 (M_age_ = 11.57, SD = 0.94) in the early pubertal, 159 (M_age_ = 12.0, SD = 1.15) in the mid pubertal, and 138 (M_age_ = 12.52, SD = 0.98) in the late pubertal stage according to their scores in the Pubertal Developmental Scale [PDS, (39, 40)]. Written informed consent was obtained from every participant and their parents prior to the experiment, and the study was approved by the Human Ethics Committee of Southwest University and Sichuan Normal University.

### Measures

#### Adolescent Developmental Stage

The pubertal developmental stages were assessed via the self-reports in the Pubertal Development Scale (PDS). The PDS is a 4-point and 5-item self-report scale that is divided into two subscales for puberty assessment in boys and girls, respectively (39, 41). The two subscales have three common items for assessing growth spurt, body hair development and skin changes in both sexes. Additionally, boys are asked to report facial hair growth and voice change, while girls report breast development and menarche [coded as 1 (not yet) or 4 (yes)]. And the internal-consistency reliability (Cronbach's alpha) of the PDS was 0.722 based on the current sample statistics (*N* = 502).

#### Depressive Symptoms

The degree of depressive symptoms was measured using the Beck Depression Inventory [BDI (42)]. BDI is a self-report questionnaire with 21 items to evaluate the presence and severity of depression in adolescents. The higher the score of the scale, the higher the degree of depression (43, 44). The highest total score was 63, which had a high convergence validity with the interviewer's score of depression severity. In this study, the Cronbach's alpha of the BDI was 0.933.

#### Perceived Stress

In this study, we measured adolescents' negative physical self, interpersonal stress, and academic self-stress.

#### Negative Physical Self

We used the Negative Physical Self Scale [NPSS (45)] to measure adolescents' negative physical self-concept. It has 48 questions divided into five dimensions, namely overall dissatisfaction, appearance, thin, short and fat, using a five-point rating scale with 0 indicating totally disagree, and 4 denoting fully agree. The average score of each dimension was obtained by averaging the total scores across all the items for each subject. The higher score denotes more dissatisfied with one's physical self. The Cronbach's alpha of each subscale based on the current sample is: α_total_ = 0.929, α_overall dissatisfaction_ = 0.701, α_fat_ = 0.914, α_thin_ = 0.817, α_appearance_ = 0.915, α_short_ = 0.904.

#### Interpersonal Stress

We used the Adolescent Interpersonal Stress Scale (AISS) to measure adolescents' interpersonal stress. The AISS has 23 items and uses a 5-point scale ranging from 0 to 4, with higher score indicating greater stress. The AISS consists of four subscales: teacher stress, peer stress, family environment stress and parental discipline stress. The scores in each subscale and the total AISS scores were computed, respectively, for each subject. The Cronbach's alpha of the AISS is: α_total_ = 0.889, α_teacher_ = 0.847, α_peer_ = 0.780, α_family environment_ = 0.542, α_parental discipline_ = 0.637.

#### Academic Self

Academic self was measured using the Adolescent Student's General Academic Self-Concept Scale (46). It has 20 items divided into four subscales: ability perception, behavioral inhibition, emotional experience and achievement value. The scale uses a 5-point scale, ranging from 1 (the description is completely unfit for me) to 5 (completely fit for me). The higher scores indicate better feeling in each specific area. The Cronbach's alpha of the scale is: α_total_ = 0.940, α_ability perception_ = 0.833, α_emotional experience_ = 0.881, α_behavioral inhibition_ = 0.867, α_achievement value_ = 0.827.

### Procedures

First, according to the predicted development level, students of grade 4–8 were randomly selected and tested with the Pubertal Development Scale (PDS). Then, the students were classified into different pubertal developmental stages according to the PDS scores. Furthermore, students of different stages and genders were asked to fill in the stress questionnaires, the Beck Depression Inventory and the Spielberger State-Trait Anxiety Inventory (STAI), with necessary guidance if needed. Finally, all the questionnaires were collected and souvenirs were distributed to the participants. Unusable questionnaires with random answers and unfinished questionnaires were deleted. A total of 502 students were randomly sampled from grade 4 to grade 8.

### Data Processing

SPSS23.0 was used for data preprocessing, analysis of variance, and calculation of correlation among various variables. Mplus7.3 was used to test each structural equation model. According to the two-step sequence of Anderson and Gerbing (47), the measurement model needs to be checked before modeling. Firstly, the measurement models of the four latent variables were tested to evaluate the goodness of fit represented by their indicators. The confirmatory factor analysis and the maximum likelihood estimation method were used to test the model fitting for the datasets, and the bias-corrected bootstrap confidence intervals were used to test the mediating effects.

The multi-item inflation measurement error of latent variables was controlled according to the packaging strategy of Wu and Wen (48). In more detail, the one-dimensional scale was packaged into three indicators by the factor balance method, and the multidimensional scale was packaged into corresponding indicators according to the number of dimensions. The mean scores of corresponding items were calculated to represent the measurement scores of the index. Since the pubertal development stage is a categorical variable, dummy coding is used for this variable in the mediation test model (49).

The following four indices were utilized to evaluate the goodness of fit of the model: (a) chi-square statistic (*χ2*), *χ2/df*, (b) the standardized root mean square residual (SRMR), (c) the root mean square error of approximation (RMSEA), and (d) the comparative fit index [CFI, (50, 51)]. According to Wen and Marsh (2004), when *χ2/df* < 5, CFI, TLI > 0.90, RMSEA < 0.08, and SRMR < 0.08, the model is considered to be well-fitted (52).

## Results

### Statistical Description and Correlation Analysis of Each Variable

Correlation analysis showed that, variables were significantly correlated with one another (Table 1).

**Table 1:** Descriptive data and zero-order correlations across variables.

	**M**	**SD**	**1**	**2**	**3**	**4**	**5**
1. PDS	5.982	2.184	1				
2. Depression	0.494	0.535	0.225^**^	1			
3. NPS	1.025	0.573	0.184^**^	0.520^**^	1		
4. IPS	0.361	0.394	0.116^**^	0.521^**^	0.401^**^	1	
5. ACS	3.619	0.817	−0.127^**^	−0.382^**^	−0.289^**^	0.304^**^	1

*PDS, Pubertal developmental stage; NPS, negative physical self; IPS, interpersonal stress; ACS, academic self*.

^*^p < 0.05,

** *p < 0.01*,

^***^p < 0.001.

**Figure 1:**
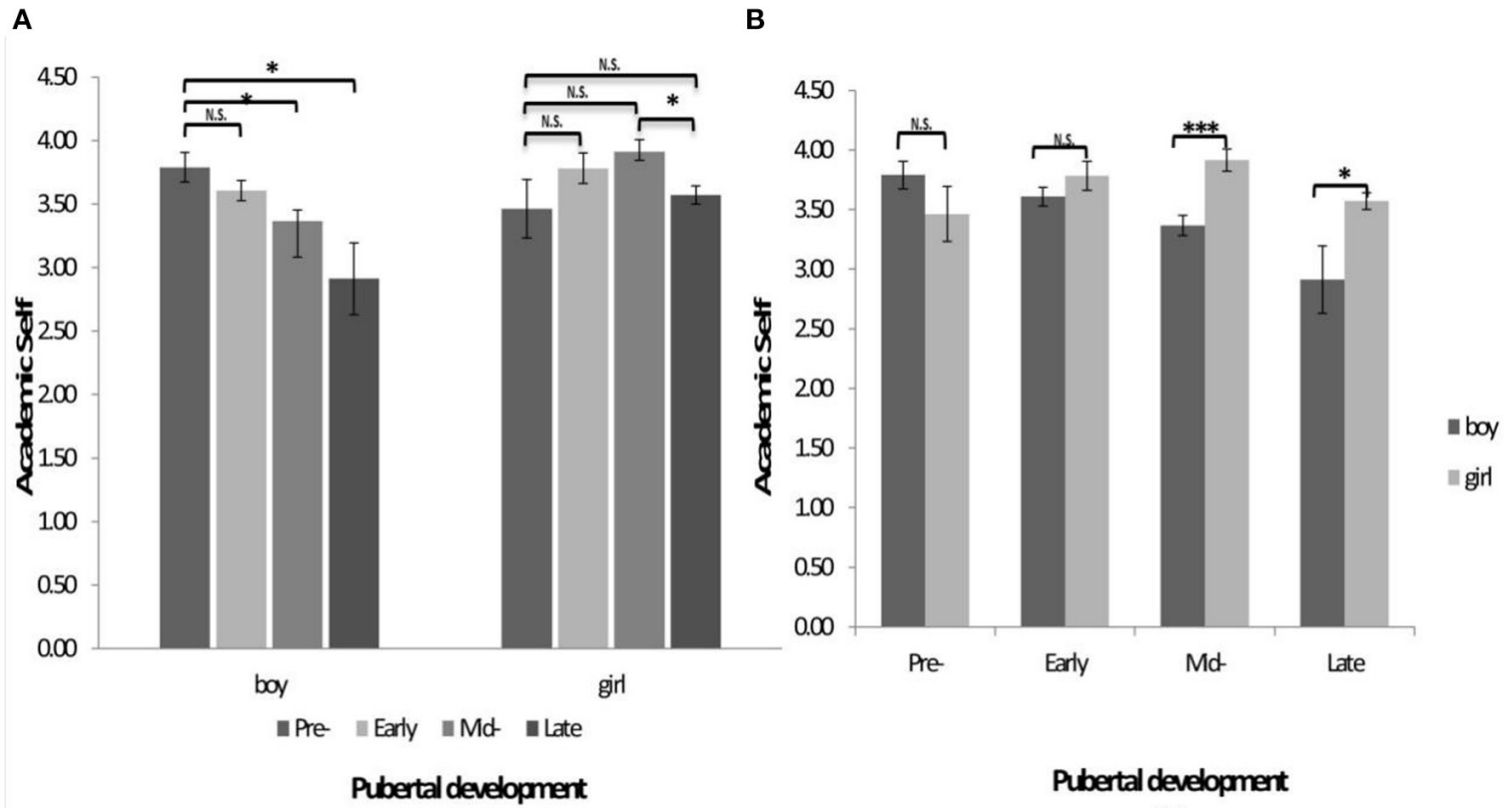
**(A,B)** The interaction of gender and pubertal development on the rating score of academic self. ^*^p < 0.05, ^**^p < 0.01, ^***^p < 0.001; N.S., not significant.

Then, the multivariate analysis of variance used gender and pubertal stage as independent variables, while depression, negative physical self, interpersonal stress and academic self as dependent variables. The results showed that the main effects of gender (*F*_(1,494)_ = 5.910, *p* < 0.05) and pubertal stage (*F*_(3,494)_ = 2.636, *p* < 0.05), as well as the gender by pubertal stage interaction (*F*_(3,494)_ = 3.956, *p* < 0.01) were significant on the academic self. The subsequent analyses showed a significant pubertal stage effect in boys (*p* < 0.01) and girls (*p* < 0.02). Boys in middle and late stages had lower academic self than in pre-pubertal stage (*p* < 0.05). For girls, the only significant comparison is the reduced academic self in late compared to the middle stage (*p* < 0.05) (Figure 1A). In another direction, boys showed lower academic self than girls in middle (*p* < 0.01) and late stage (*p* < 0.05) but not in the pre-and early stage (*p* > 0.1) (Figure 1B).

In addition, there was a significant main effect of pubertal stage on depression *F*_(3,494)_ =4.288, *p* < 0.01. Post *hoc* comparisons showed that the depression level in late stage was significantly higher than that in pre- (*p* < 0.01), early- (*p* < 0.01), and mid-stages (*p* < 0.01), while no significant differences were observed amongst pre-, early and mid-stages (*p* > 0.1). There were no other significant main or interaction effects.

### Measurement Model

Confirmatory factor analysis was used to test whether the measurement model could adequately fit the sample data. The full measurement model included four latent constructs (depression, interpersonal stress, academic self and negative physical self), which altogether generated sixteen observed variables. The results showed that the measurement model can fit the sample data well (*χ2* = 330.093, *df* = 98, *χ2/df* = 3.368, CFI = 0.942, TLI = 0.929, RMSEA = 0.069, SRMR = 0.068). However, among the five indicators of negative physical self, the factor loading coefficients of the overall dissatisfaction and thin were lower than 0.45 (0.281 and 0.399, respectively), so these two indicators were deleted (52) to improve the model fitting (*χ2* = 170.894, *df* = 71, *χ2/df* = 2.410, CFI = 0.973, TLI = 0.966, RMSEA = 0.053, SRMR = 0.046). Then, the standardized factor loadings of all the observed variables on the corresponding latent constructs were statistically significant (*p* < 0.001), indicating that the structural equation model can be used in the next step of analysis.

### Common Method Bias Test

Using Harman single factor test, we analyzed the principal component factors of all the items in the questionnaire without rotation. The results showed that the variance of the maximum factor interpretation was 15.91% (<40%), which indicated that the common method bias of this study was not significant (53).

### Structural Model

In the first step, as mentioned above, the direct effect of the predictor (pubertal stage) on the dependent variable (depression) without mediators was significant (*F*_(3,498)_ =4.288, *p* < 0.01). The level of depression tended to increase as a function of pubertal stage (pre: 0.384 ± 0.469, early: 0.425 ± 0.506, mid: 0.436 ± 0.507, late: 0.684 ± 0.581), and depression was significantly higher in the late compared to other pubertal stages. Therefore, the pre puberty was used as the reference baseline, and three dummy variables of early, middle and late puberty were set as independent variables. Then, Model 1 and Model 2 were established with negative physical self (M1) or interpersonal stress (M2) as mediators. Model 3 was established with academic self as mediator and gender as moderator, given observation of a gender & puberty interaction effect on academic self. The results of the mediation model showed a satisfactory fitting of Model 1, Model 2 (Model 1: *χ2* = 60.195, *df* = 20, *χ2/df* = 3.010, CFI = 0.977, TLI = 0.962, RMSEA = 0.063, SRMR = 0.033, Model 2: *χ2* = 42.004, *df* = 28, *χ2/df* = 1.500, CFI = 0.993, TLI = 0.989, RMSEA = 0.032, SRMR = 0.023).

**Figure 2:**
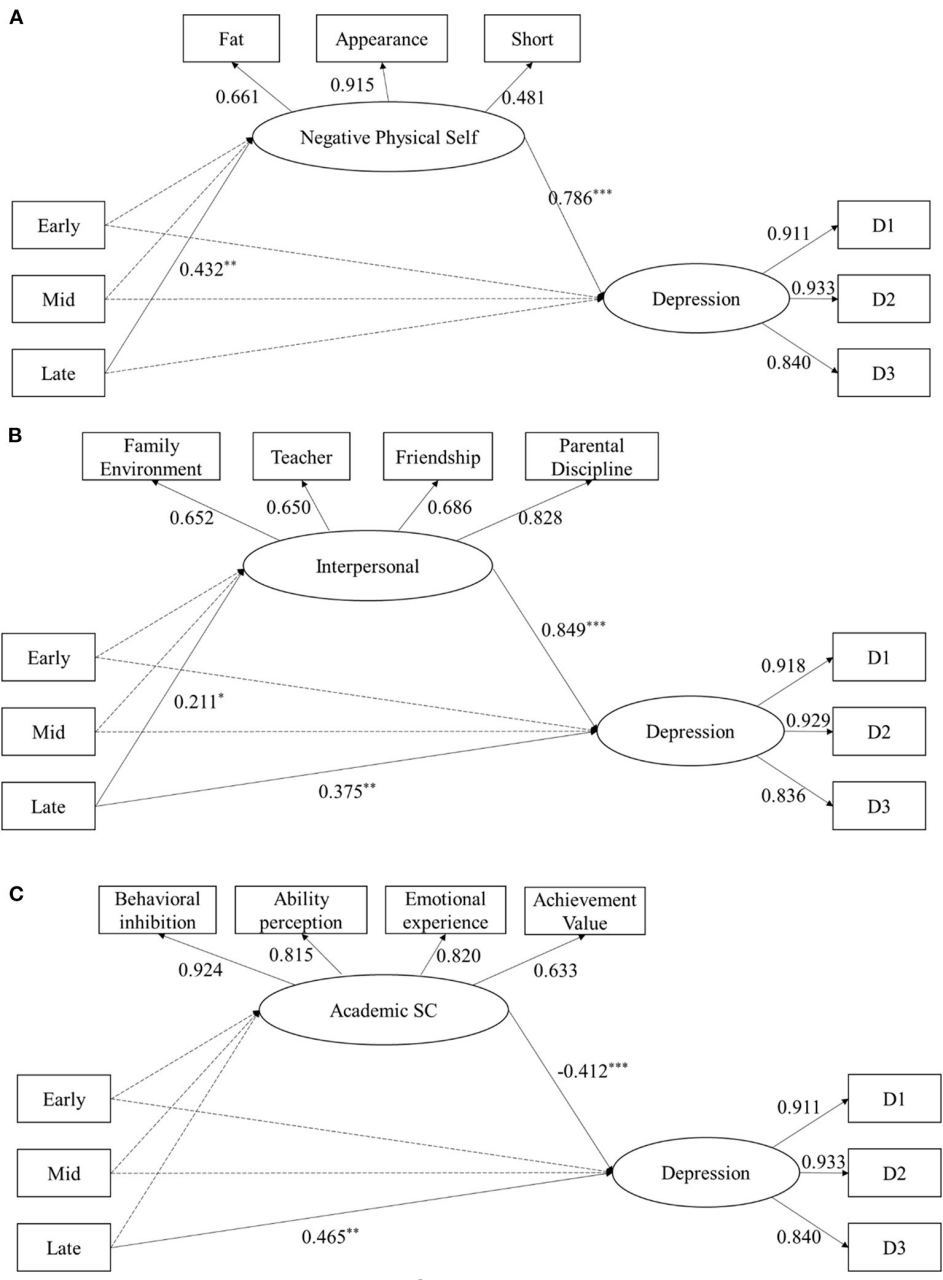
Single factor mediation Model 1, Model 2 and Model 3 were established with negative physical self **(A)** or interpersonal stress **(B)** as mediator, respectively. Model 3 was established with academic self **(C)** as mediator and gender as moderator. Path coefficient was standardized. ^*^p < 0.05, ^**^p < 0.01, ^***^p < 0.001, Early, Early puberty; Mid, Mid puberty; Late, Late puberty.

The bias-corrected bootstrap method was used to test the mediating effect (sampling = 1000). The results showed that the mediating effects of Model 1 and Model 2 were not significant in the early and mid-stage, while the mediating effects of negative physical self (Model 1) and interpersonal stress (Model 2) were significant in the late stage (Figures 2A,B). The mediating effect of academic self (Model 3) was not significant (Figure 2C). Monte Carlo power analyses were used to calculate the statistical power of Model 1 and Model 2. Results showed that Model 1 (negative physical self) had a statistical power of 0.99, while Model 2 (interpersonal stress) had a statistical power of 0.74.

**Figure 3:**
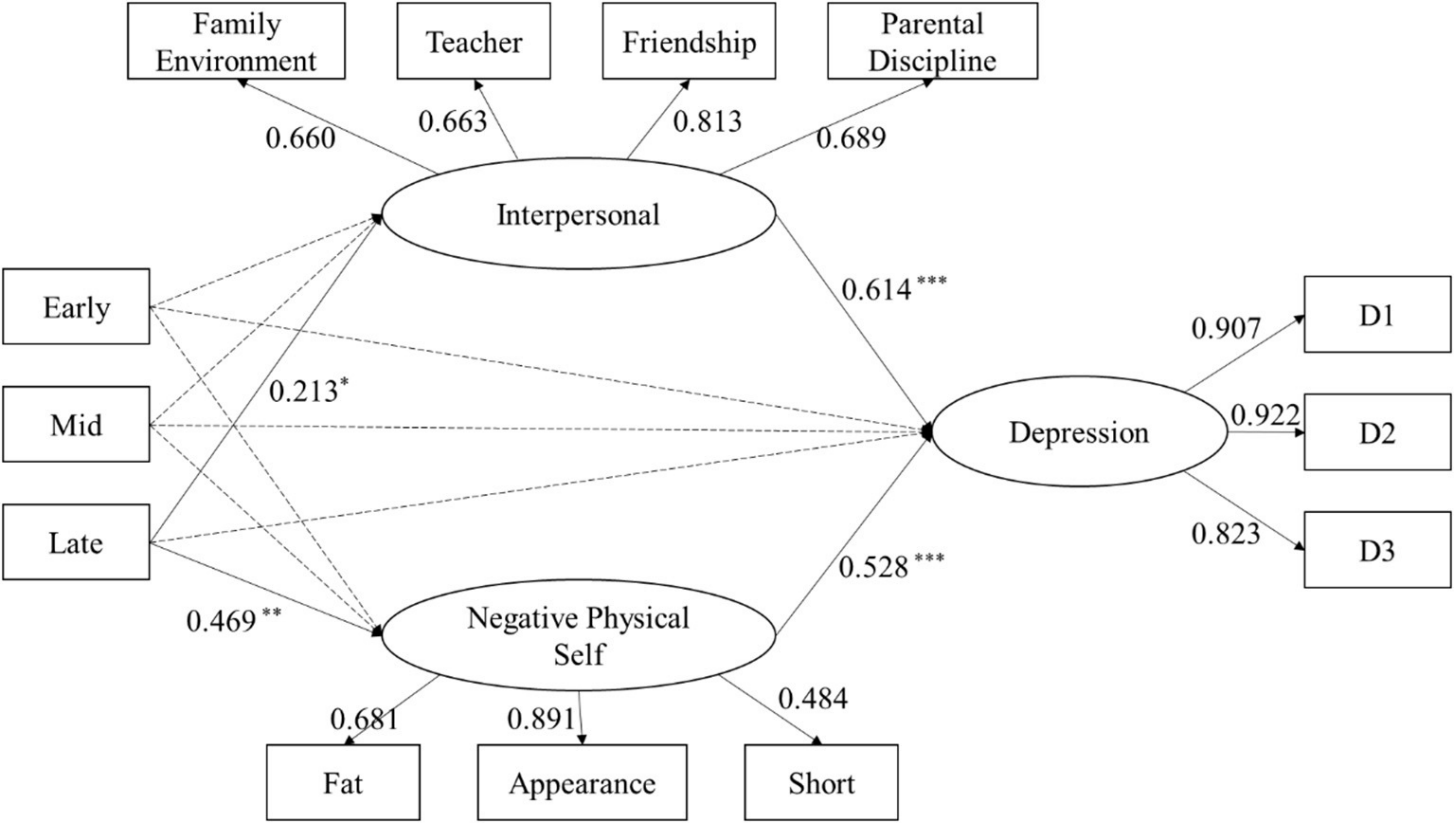
Multiple mediation Model 4 between the pubertal stage and depression through interpersonal stress and negative physical self. Path coefficient was standardized. ^*^P < 0.05, ^**^P < 0.01, ^***^P < 0.001; Early, Early puberty; Mid, mid puberty; Late, late puberty.

According to the results of the single factor mediation model, the multiple mediation model was established with negative physical self and interpersonal stress as mediators (Model 4). The model fitting is satisfactory, *χ2* = 210.420, *df* = 54, *χ2/df* = 3.896, CFI = 0.939, TLI = 0.915, RMSEA = 0.076, SRMR = 0.092. In Model 4, the mediating effect of interpersonal stress was not significant in the early and mid-puberty. The mediating effects of interpersonal stress [95% CI: (0.002, 0.283)] and negative physical self [95% CI: (0.134, 0.431)] were significant in the late pubertal stage (Figure 3).

## Discussion

This research yielded three main results. First, through correlation analysis, we found that pubertal development is associated with an increase in the levels of depression and psychosocial stresses in adolescents. Specifically, the level of depression, psychosocial stresses such as negative physical self, interpersonal stress and perceived academic stress all increased with pubertal development. In addition, multiple psychosocial stressors were positively correlated with the level of depression, which implies that the stresses coupled by puberty development may contribute to the generation of depression. Second, concerning the gender differences in depression and stressors across pubertal stages, we only observed a significant pubertal stage and gender interaction effect on the ratings of academic self. Boys, instead of girls, faced higher academic stress with pubertal development, while the analysis of other stressors (interpersonal stress and negative physical self) did not show this pattern of effect. Moreover, our correlation analyses showed greater stresses in the measures of academic self, negative physical self and interpersonal stress with pubertal development. Third and most important, single factor mediation analysis showed that negative physical self and interpersonal stress mediates the increased depression in late compared to pre pubertal stages, respectively. The significant mediators of interpersonal stress and negative physical self are further replicated in the multiple-factor mediation model.

### The Gender and Puberty Interaction on Academic Self-Concept

Our finding of higher academic stress in boys but not in girls with pubertal development is consistent with a couple of prior observations. For example, there was evidence showing an advantage of girls over boys in the four dimensions of the academic self (54–56). First of all, research has shown that girls' academic performance is generally better than boys around the senior grades of primary school or early high school (56). Also, early study has shown that girls are more inclined than boys to internally attribute improved academic performance to their own efforts and abilities through positive perception (55). In addition, girls outperform boys in behavioral self-control (57). On the other hand, boys have more academic procrastination than girls, like delayed implementation and delayed remediation (54), and boys experience more negative academic emotions than girls.

The results suggest that the effects of pubertal development on negative physical self, interpersonal stress, and depression were consistent across boys and girls. One explanation for the lack of gender differences in these measures may be related to the range of ages of the subjects (10–14 years). Early studies have shown that there is no gender difference in depressive mood for adolescents at 12–14 years old (58), while gender differences appear from 14 to 15 years (14, 59). This coincided with our finding of rising depression in late compared to pre, early and mid-pubertal stages, while the latter three stages showed similarly low levels of depression across genders.

### The Influence of Pubertal Development on Adolescent Depression via Negative Physical Self and Interpersonal Stress

Previous studies have shown that body dissatisfaction was a major predictor of low self-esteem, depression, and eating disorders in adolescents (60–62). In addition, the concept of “thinness as beauty for girls and strong as beauty for boys” usually prevails, leading to teenager's irrational expectations for figure, weight and increased physical self-dissatisfaction since entry into puberty. For example, it has been reported that stress from peers, parents and social media predisposes teenagers to be easily dissatisfied with body image, thus promoting the risk of depression (27, 63).

On the other hand, adolescents have excessive comfort seeking and greater emotional dependence on their friends compared to people in other periods (64), which suggests that adolescents are more susceptible to interpersonal stress (e.g., relationship breakdown) than other age groups, which also increases the risk of depressive symptoms (64). Recent studies have shown that multiple physiological indicators (e.g., salivary alpha amylase, systolic blood pressure) of adolescents are significantly higher than those of children during peer rejection or social exclusion (64–66). In addition, parent-child conflict constitutes an important part of interpersonal stress at adolescence, since seeking affective and behavioral independence from parents is one of the key motivations in this period (30, 67).

## Conclusion and Future Directions

In summary, the current study identified negative physical self as a key mediator underpinning the predictive role of puberty in adolescent depression. This shows that negative physical self is a major stressor that contributes to adolescent depression during late pubertal period. However, the current cross-sectional design measured depression, pubertal stage and psychosocial stressors at the same phase. In this regard, though we observed reliable mediation effects of negative physical self and interpersonal stress on the association between puberty and depression, the causal relationship between adolescent depression, pubertal stage and psychosocial variables cannot be firmly inferred. Therefore, future studies need to administer a longitudinal design to a large sample of subjects across different pubertal stages, to determine the influence of puberty on affective health and its related psychosocial mechanisms.

## Data Availability Statement

The original contributions presented in the study are included in the article/supplementary material, further inquiries can be directed to the corresponding authors.

## Ethics Statement

The studies involving human participants were reviewed and approved by Ethics committee of human research at Southwest University and Sichuan Normal University. Written informed consent to participate in this study was provided by the participant's legal guardian/next of kin.

## Author Contributions

JY: conceptualized the study and research design. JY and DY: paper writing. LJ: data analysis and presentation. DY and YL: data connection. All authors contributed to the article and approved the submitted version.

## Funding

This study was supported by the National Natural Science Foundation of China (NSFC31971018; 31871103).

## Conflict of Interest

The authors declare that the research was conducted in the absence of any commercial or financial relationships that could be construed as a potential conflict of interest.

## Publisher's Note

All claims expressed in this article are solely those of the authors and do not necessarily represent those of their affiliated organizations, or those of the publisher, the editors and the reviewers. Any product that may be evaluated in this article, or claim that may be made by its manufacturer, is not guaranteed or endorsed by the publisher.
